# ARE/SUZ12 dual specifically-regulated adenoviral TK/GCV system for CML blast crisis cells

**DOI:** 10.1186/s13046-015-0139-4

**Published:** 2015-05-28

**Authors:** Bailing Zu, Yi Shi, Min Xu, Guoling You, Zhenglan Huang, Miao Gao, Wenli Feng

**Affiliations:** Department of Clinical Hematology, Key Laboratory of Laboratory Medical Diagnostics of Ministry of Education, Chongqing Medical University, No.1, Yixueyuan Road, Chongqing, 400016 People’s Republic of China; Department of Clinical Laboratory, Shanghai Pudong Hospital, Fudan University Pudong Medical Center, Shanghai, China; Department of Clinical Laboratory, Shanghai Children’s Medical Center, Shanghai Jiaotong University School of Medicine, Shanghai, China

**Keywords:** Suicide gene, Gene therapy, Chronic myelogenous leukemia, Blast crisis, Gene regulation

## Abstract

**Background:**

Treatment of blast phase chronic myeloid leukemia (BP-CML) remains a challenge, and the median survival is less than 6 months. Because effective treatments are lacking, we studied tight targeting of blast crisis CML cells using adenoviral (Ad) vectors expressing a HSV-TK system under dual control of a specific SUZ12 promoter and an antioxidant response element (ARE).

**Methods:**

A potential SUZ12 promoter fragment was designed with bioinformatics databases and identified with a luciferase assay. Next, we cloned the ARE element of the *NQO1* gene and developed Ad vectors expressing TK kinase or luciferase under the dual control of a specific SUZ12 promoter and an ARE element. An *in vitro* transfection assay with Ad-ARE/SUZ12-Luc was used to determine promoter activity of ARE/SUZ12 regulatory element in blast crisis CML cells. After incubating human BP-CML-derived cells with Ad-ARE/SUZ12-TK and ganciclovir, Western blot, CCK8, Immunofluorescent assays and Annexin V assays were conducted to assess the efficacy of an ARE/SUZ12 dual-specific TK/GCV system for BP-CML cell lines.

**Results:**

Here, luciferase data confirmed significantly higher and specificer promoter activity of the ARE/SUZ12 composite component in CML blast crisis-derived cell lines (K562, KCL22, and K562/G01) compared to HepG2 cells, and Ad-AS-TK/GCV system could exhibit enhanced apoptotic effects and decreased cell viability for BP-CML cell lines. Additionally, Ad-AS-TK/GCV system altered expression of cycle-related and apoptosis-related proteins in BP-CML cell lines.

**Conclusions:**

Thus, ARE/SUZ12 dual targeting TK/GCV system was effective in killing BP-CML cells. Moreover, efficacy and specificity of CML cell eradication were enhanced by synergistic effects of ARE/SUZ12 dual-specific regulation. We conclude that suicide gene-targeted therapy might hold promise for BP-CML treatment.

**Electronic supplementary material:**

The online version of this article (doi:10.1186/s13046-015-0139-4) contains supplementary material, which is available to authorized users.

## Background

Chronic myeloid leukemia (CML) is a clonal myeloproliferative disorder of hematopoietic stem cells, accounting for 20% of newly diagnosed adult leukemia cases. The natural history of CML is a stereotypical progression from the chronic phase (CP), through progressive deterioration to a final fatal blast crisis phase (BP) [1]. The introduction of tyrosine kinase inhibitors (TKIs) has dramatically altered CP-CML outcomes [2], improving 8-year overall survival (OS) from 20 to 85%; however, BP-CML remains a challenge, with a 75% mortality within six months. Until now, BP-CML treatment has lacked a therapeutic agent, and allogeneic stem cell transplantation (alloSCT) has been considered as the only curative option for BP-CML, even though post-transplantation prognosis is poor (2-year OS rate of 16%) [3]. To overcome limitations of current therapy methods, gene therapy has been suggested to be as a potential novel approach for BP-CML.

Suicide gene therapy has been proved non-invasive and effective for ovarian cancer, central nervous system tumors, and graft-versus-host disease (GVHD) syndrome treatment [4–6]. Among suicide gene therapy strategies, the herpes simplex virus thymidine kinase gene (HSV-Tk)/ganciclovir (GCV) system is the most commonly used for tumor treatment research. Basically, a suicide gene strategy is based on expressed enzymes of transgenes, which convert a non-toxic prodrug into a toxic antimetabolite, and then HSV-TK converts GCV into a toxic phosphorylated form that subsequently induces apoptosis by inhibiting DNA synthesis and blocking the cell cycle. Additionally, a distant bystander effect of the TK/GCV system, the regression of tumors distant from those cells expressing TK, occurs *in vivo* mainly via immune stimulation. Therefore, the TK/GCV suicide gene system is a powerful strategy for cancer gene therapy.

SUZ12 is a core component of polycomb repressive complex 2 (PRC2), essential for regulation of embryogenesis, tissue development, and stem cell self-renewal. SUZ12 is both highly and specifically expressed in stem cells, and rapidly down-regulated during differentiation. In normal human tissues, SUZ12 expression is restricted to proliferating cells in reactive lymphoid tissue, germinal cells in the testis and the epithelium of various organs [7]. Recently, studies have shown that substantial SUZ12 expression can promote cell proliferation, inhibit cell differentiation, disrupt histone modification dynamics and, consequently, induce tumorigenesis [8–11]. Moreover, high promoter activity of the *SUZ12* gene have been shown to be involved with upregulated SUZ12 expression and colon or liver tumor progression [12]. Pizzatti’s group reported high promoter activity of SUZ12 in BP-CML mononuclear cells but a lack of SUZ12 expression in normal counterparts, because the noncanonical WNT pathway activates the SUZ12 promoter in BP-CML cells [13,14].

CML is a clonal myeloproliferative disorder resulting from a balanced translocation between the long arms of chromosomes 9 and 22, which generates the hybrid *BCR-ABL* gene on chromosome 22q. The product of the *BCR-ABL* oncogene is a bcr-abl protein which has high tyrosine kinase activity. As a potent kinase, BCR-ABL constitutively produces intracellular ROS [15,16], which activates the nuclear factor E2-related factor 2 (Nrf2) pathway. High intracellular ROS in BP-CML activate Nrf2 to recognize the antioxidant response element (ARE) of antioxidant responsive genes and drive expression of nuclear ARE-regulated genes. In normal cells under homeostatic conditions, Nrf2 is generally localized to the cytoplasm and is constantly degraded via Keap1-mediated ubiquitination, thus, the ARE element of downstream genes was silenced.

In this study, different potential SUZ12 promoter sequences were cloned based on bioinformatic predictions and identified by luciferase assay. Then, we constructed a BP-CML cell-specific regulatory element composed of a SUZ12 promoter and an ARE element from the *NQO1* gene. Finally, we explored the feasibility of combining the SUZ12 promoter and an ARE regulatory element to target BP-CML cells with high specificity. Although prospective results of gene therapy have been reported [17], strategies are needed to overcome current gene therapy issues such as non-specific normal cell death and cytotoxicity. Here, we report that ARE/SUZ12-regulated HSV-TK/GCV therapy is effective and specific for BP-CML cells *in vitro*, and this may offer an attractive therapeutic approach for treating BP-CML in the future.

## Methods

### Cell culture

Human BP-CML derived K562, KCL22 and imatinib-resistant K562/G01 cell lines were maintained in RPMI-1640 medium (Sigma, USA) supplemented with 10% (v/v) FBS (Sigma), 100 U/ml penicillin, and 100 μg/ml streptomycin. Ad293, Phoenix-Ampo and HepG2 cell lines were maintained in Dulbecco’s Modified Eagle’s Medium (Sigma) supplemented with 10% (v/v) FBS, 100 U/ml penicillin, and 100 μg/ml streptomycin. Cell cultures were maintained at 37°C under 5% CO_2_.

### Bone marrow samples

Bone marrow samples were obtained from CML patients and healthy donors admitted or registered at the first Affiliated Hospital of Chongqing Medical University (Chongqing, China), according to the guidelines of the local Ethics Committee. Bone marrow mononuclear cells were isolated from 2-ml aspirates using Ficoll gradient (Tianjin Haoyang Biological Manufacture Co., China). This study was approved by the Ethics Committee of Chongqing Medical University and a written informed consent was obtained from all participants.

### Semi-quantitative RT-PCR and quantitative real-time PCR analysis (qRT-PCR)

Cells were collected for RNA extraction with RNAiso Plus (Takara, China). RNA concentration and purity was confirmed by OD_260/280_ readings, and 1 μg of RNA was reverse transcribed into cDNA using a PrimeScript® RT reagent Kit with gDNA Eraser (Takara). Semi-quantitative PCR amplifications were performed with Taq polymerase (Takara) on Bio-Rad T100™ Thermal Cycler (Bio-Rad, USA), and gene expression was compared to β-actin (control), by agarose gel electrophoresis. Real time PCR (qPCR) was performed using an SYBR premix Ex Taq™ kit (Takara) on a Bio-Rad CFX96 Real-time PCR detection System (Bio-Rad, USA). Then, samples were transferred to a thermal cycler and DNA was amplified through 40 thermal cycles under the following conditions: denaturation at 95°C for 10 s, annealing at 55°C for 20 s, and extension at 72°C for 20 s. PCR primers used were as follows: 5’-CCGAGCACTGTGGTTGAGTA-3’ (forward) and reverse 5’-AACTGCATCTGATGGTGGTG-3’ (reverse) for SUZ12,5’-CAGCCAGCCCAGCACAT-3’ (forward) and 5’-AGCCGAAGAAACCTCATTGTC-3’ (reverse) for Nrf2, and forward 5’-CTGAAGTACCCCATCGAGCACGGCA-3’(forward) and 5’-GGATAGCACAGCCTGGATAGCAACG-3’(reverse) for β-actin.

### SUZ12 promoter analysis

Genomic DNA of K562 cells was extracted with a Wizard® Genomic DNA Purification Kit (Promega, USA), and then the 2,264, 1,375, and 793 bp upstream region fragments of the *SUZ12* gene were amplified by PCR from K562 genomic DNA. 5’-CTCAGAGGATTGGTGTGAGAATTGAATGT-3’, 5’-ATGGTTCTATGTTTATAGGTTGGG-3’, and 5’-TGACCACTCTTACGGTGTTTG-3’ were sense primers of the three nucleotide fragments, respectively. The anti-sense common primer of the three nucleotide fragments was 5’-GCCTGGACGTGCTCCATTTTCG-3’.

The three fragments were inserted into promoter-less vectors pGL3-Basic (Promega), named pGL3-S1, pGL3-S2, and pGL3-S3, respectively, and the identity of all the three constructs was confirmed by DNA sequencing. Next, plasmids and Mig-p210 were co-transfected into Phoenix-Ampo cells using JetPEI (Polyplus, France), moreover, to exclude the transfection efficiency factor, promoter activity analysis was conducted with pRL-TK (Promega). Cells were harvested 48 h later and assayed with a dual-luciferase reporter assay system (Promega).

### Adenovirus vector construction, production, and transduction

A SUZ12 promoter, CMV minimal core promoter, and HSV-TK encoding sequences were obtained by PCR from pGL3-S1, plasmid pIRES2-EGFP (Clontech, USA) and pORF9-HSV1tk (Invitrogen, LOCATION), respectively. The ARE domain was chemically synthesized and cloned into pAdTrack (Invitrogen) by denaturing and annealing. PCR primers used were as follows: 5’-CTCAGAGGATTGGTGTGAGAATTGAATGT-3’ (forward) and 5’-GCCTGGACGTGCTCCATTTTCG-3’ (reverse) for SUZ12, 5’-ACGCGTCGACAGAATTACACGGCGATCTTTC-3’ (forward) and 5’-AGAATTACACGGCGATCTTTC-3’ (reverse) for Luc, forward 5’-GCCACCATGGCCTCGTA-3’ (forward) and 5’-TCAGTTAGCCTCCCCCATCTC-3’ (reverse) for TK, forward 5’-AGCTTATCCGCAGTCACAGTGACTCAGCAGAATCTGATCCGCAGTCACAGTGACTCAGCAGAATCTGC-3’ (forward) and 5’-TCGAGCAGATTCTGCTGAGTCACTGTGACTGCGGATCAGATTCTGCTGAGTCACTGTGACTGCGGATA-3’ (reverse) for ARE, forward 5’-TAGGCGTGTACGGTGGGAGGTCTAT-3’ (forward) and 5’-GGATCTGACGGTTCACTAAACCAG-3’ (reverse) for CMV.

**Figure 1:**
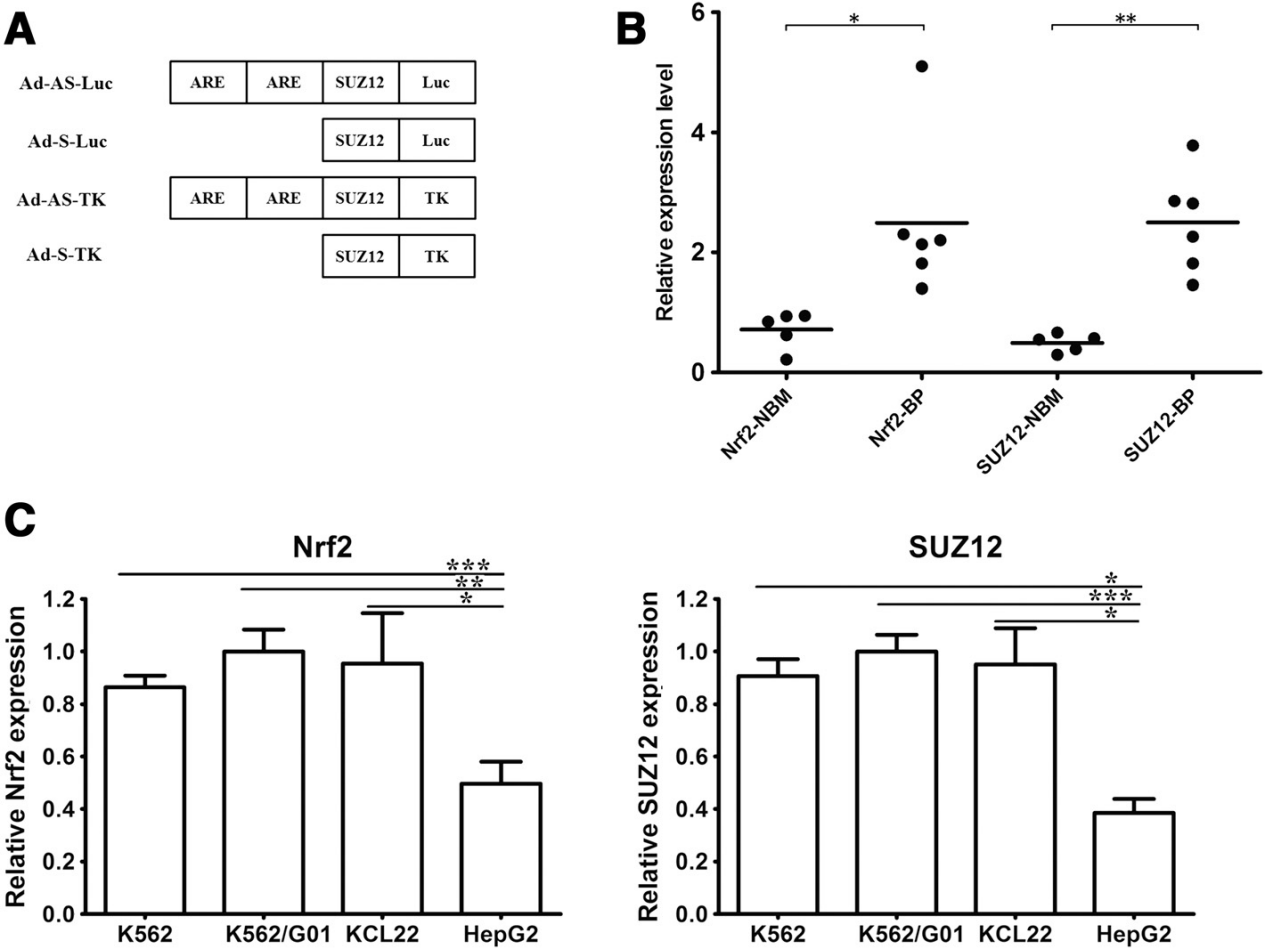
SUZ12 and Nrf2 status in blast crisis CML cells **(A)** Structures of Ad luciferase reporter (Ad-AS-luc and Ad-S-luc) and Ad TK vectors (Ad-AS-TK and Ad-S-TK). The ARE construct contains 2 AREs from human NQO1 modifier subunit, and the SUZ12 promoter contains a 2.3 kb upstream fragment. **(B)** Relative SUZ12 and Nrf2 mRNA of mononuclear cells from five normal bone marrow and six blast phase samples, by RealTime RT-PCR. NBM: normal bone marrow; BP: blast phase. **(C)** Relative SUZ12 and Nrf2 mRNA in K562, KCL22, K562/G01, and HepG2 cells by RealTime RT-PCR analysis. Data are depicted as relative to actin mRNA, respectively. *, P < 0.05; **, P < 0.01; ***, P < 0.001.

DNA sequences encoding HSV-TK or luciferase, the SUZ12 promoter sequence, the CMV minimal core promoter, and the ARE domain were linked into DNA cassettes (Figure 1A). Recombinant replication-deficient adenoviruses were produced using the AdEasy system as previously described [18]. For Ad transduction, different multiplicities of infection (MOI) that yielded similar transduction efficiency for the four cell lines (~80%) were used. K562, KCL22, K562/G01 were incubated with adenoviruses (MOI 500:1) and polybrene (Sigma, USA) at 10 μg/mL, while HepG2 was incubated with adenoviruses at an MOI of 50:1.

### Ad luciferase assay

For the Ad luciferase assay, cells were seeded (1 × 10^4^ cells/well) into 24-well plates, and transduced with Ad-AS-luc, Ad-S-luc, or Ad-control as described above. After 72 h, a luciferase assay was performed with a ONE-Glo™ Luciferase Assay System (Promega) according to the manufacturers’ instructions. Results were normalized to protein measured with the Bradford method and all data were depicted relative to luciferase activity of Ad-control. Luciferase of Ad-control was driven by the CMV minimal core promoter, whereas Ad-AS-luc and Ad-S-luc were driven by ARE/SUZ12 and SUZ12, respectively.

### Cell survival assay

To measure the effects of TK/GCV on BP-CML cell viability, cells were seeded (2 × 10^3^ cells/well) into 96-well plates, and transduced with Ad-AS-TK, Ad-S-TK, or Ad-Empty as described above, and then 24 h later, 0–100 μmol/l GCV was added to the medium. After 48 h of GCV incubation, medium was replaced with fresh medium containing GCV. After 0, 24, 48, 72, 96, and 120 h of GCV incubation, cell viability was measured with a WST assay using a cell counting kit (CCK) (Beyotime, China), and survival was calculated according to relative absorbance at 450 nm.

### Immunofluorescent assays

Transduced cell cells were fixed in 4% (v/v) paraformaldehyde for 20 min, permeabilized for 15 min in PBS containing 0.1% (v/v) Triton X-100, and then preblocked for 1 h in 1% (w/v) goat serum (Sigma) at room temperature. Cells were incubated overnight at 4°C with primary antibodies against SUZ12 (1:350, Cell Signaling, USA), NQO1 (1:200, PTG, USA). Secondary antibodies (1:1,000, anti-rabbit or anti-mouse, Santa Cruz, CA) were added and incubation proceeded at room temperature for 1 h in the dark. Nuclei were counterstained with DAPI (1:1,000, Sigma).

### Western blot

To measure expression of cyclical and apoptotic signaling molecules, cells was seeded (2 × 10^5^ cells/well) into 6-well plates, and transduced with Ad-AS-TK, Ad-S-TK, or Ad-Empty and 100 μmol/l of GCV as described above. After 48 h of GCV incubation, cells were lysed in RIPA lysis buffer. Cell protein was quantified using the Bradford method, separated by 10% SDS-PAGE, electrotransferred to PVDF membranes, and then immunoblotted with primary antibody against Bcl-2, Bax, and cyclin D1 (Cell Signaling), followed by HRP-conjugated secondary antibody. Protein band intensity was quantified via ECL (Millipore, USA) with a Viagene Cool Imager™ (Viagene, USA).

### Apoptotic analysis

To measure apoptosis, cells were seeded (2 × 10^5^ cells/well) into 6-well plates, and transduced with Ad-AS-TK, Ad-S-TK, or Ad-Empty as described above, and further incubated with 100 μmol/l of GCV. After observing apoptosis with Wright’s staining, apoptosis was measured with PE Annexin V (BD Biosciences, USA), according to the manufacturer’s instructions, followed by cell counting with a Gallios flow cytometer (Beckman Coulter, USA).

### Statistical analysis

All experiments were conducted at least three times, and data are expressed as means ± SD. Statistical differences between groups were measured with a Student’s *t*-test, and statistical differences across cell types and Ad types were measured with a 2-way ANOVA *post hoc* test (P <0.05 were considered statistically significant).

## Results

### Status of SUZ12 and Nrf2 in BP-CML

mRNA of both SUZ12 and Nrf2 was measured in three BP-CML-derived cell types (K562, KCL22, and K562/G01), and a HepG2 hepatoma cell line. Figure 1C shows statistically significant differences of SUZ12 and Nrf2 mRNA between BP-CML cell lines and HepG2 (P <0.05), SUZ12 and Nrf2 mRNA were both significantly higher in the CML blast crisis-derived cell lines compared to HepG2 cells. To measure SUZ12 and Nrf2 mRNA in bone marrow mononuclear cells from five healthy donors and six blast phase patients, we used qRT-PCR and observed significant increases in SUZ12 and Nrf2 in blast crisis samples compared to normal donor samples (P <0.05). These data agreed with previous published reports [14,19].

### SUZ12 and ARE/SUZ12 promoter activity in different cell lines

**Figure 2:**
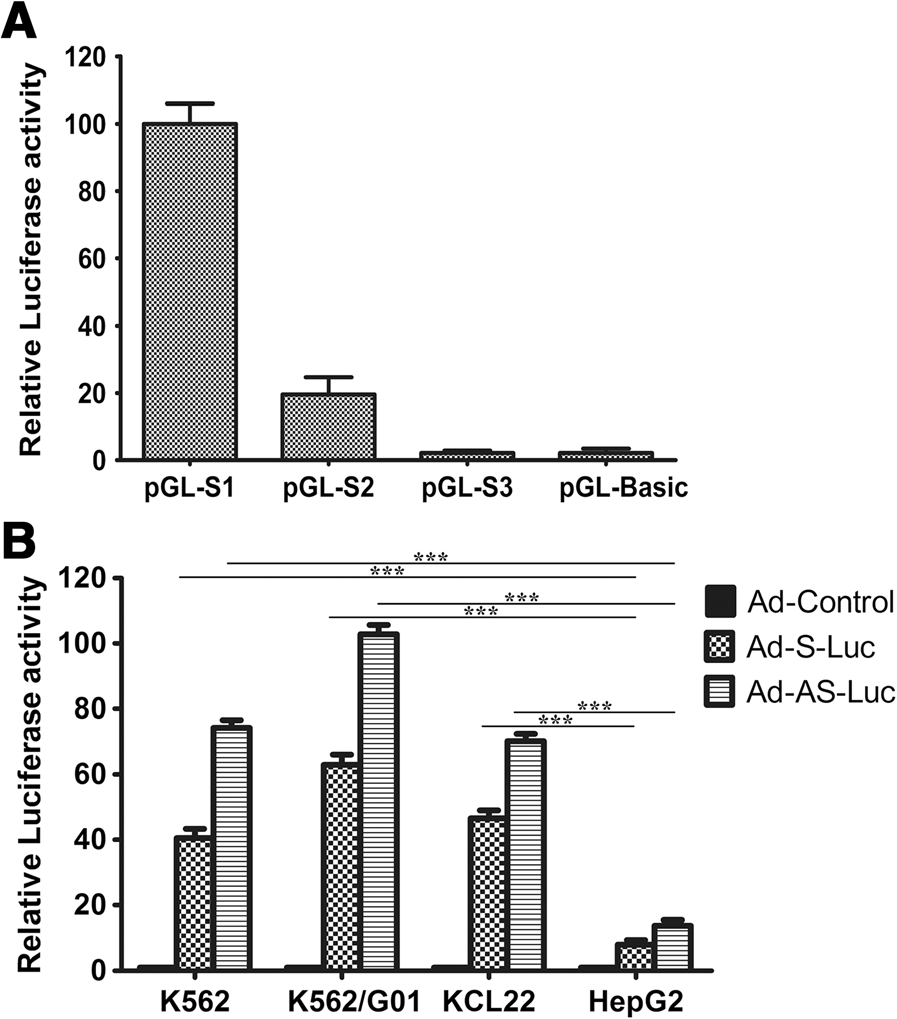
Basal and inducible SUZ12 activity in Bcr/abl^+^ cells. **(A)** Promoter activity assay of different SUZ12 upstream fragments via dual-luciferase reporter assay in Bcr/abl^+^PHA cells. Bcr/abl^+^PHA cells were transfected as depicted in Methods and luciferase activity was measured 48 h later. Data are presented as a fold-changes ± SD of firefly/*Renilla* (F/R) ratios, means ± SEM, n=3. **(B)** Promoter activity assay of SUZ12 or ARE/SUZ12 by ONE-Glo™ luciferase assay in K562, K562/G01, KCL22, and HepG2. Cells were transduced as depicted in Methods and luciferase activity was measured 72 h later. Data are normalized to protein and are presented as fold-increases versus Ad-control, means ± SEM, n=3. *, P < 0.05; **, P < 0.01; ***, P < 0.001.

Using bioinformatics and the literature [12], the 2,264 bp (−1,793 to +471), 1,375 bp (−904 to +471), and 793 bp (−322 to +471) upstream fragments of the *SUZ12* gene were inserted to pGL3-basic reporter plasmids, and luciferase activity of the constructed reporter plasmids was measured in Phoenix-Ampo/Mig-p210 cell to confirm the active promoter sequence. Promoter activity of the 2.3 kb construct was approximately four times greater than the 1.4 kb fragment; thus the distal region of the SUZ12 promoter had more basal promoter activity under control conditions (Figure 2A).

Next, we measured basal SUZ12 or ARE/SUZ12 promoter activity in K562, KCL22, K562/G01, and HepG2 cells by transducing cells with the Ad-control, Ad-S-luc or Ad-AS-luc (construct of Ad-control, Ad-S-luc, or Ad-AS-luc, see §Materials and Methods). Luciferase assay data indicated that K562/G01 had the most ARE/SUZ12 activity, and this was 102-fold greater than the Ad-control, whereas HepG2 had the least activity (Figure 2B). Also, data confirmed significantly higher and specificer promoter activity of the ARE/SUZ12 composite component in CML blast crisis-derived cells (p < 0.05).

### Viability of BP-CML cells transduced with Ad-AS-TK or Ad-S-TK

**Figure 3:**
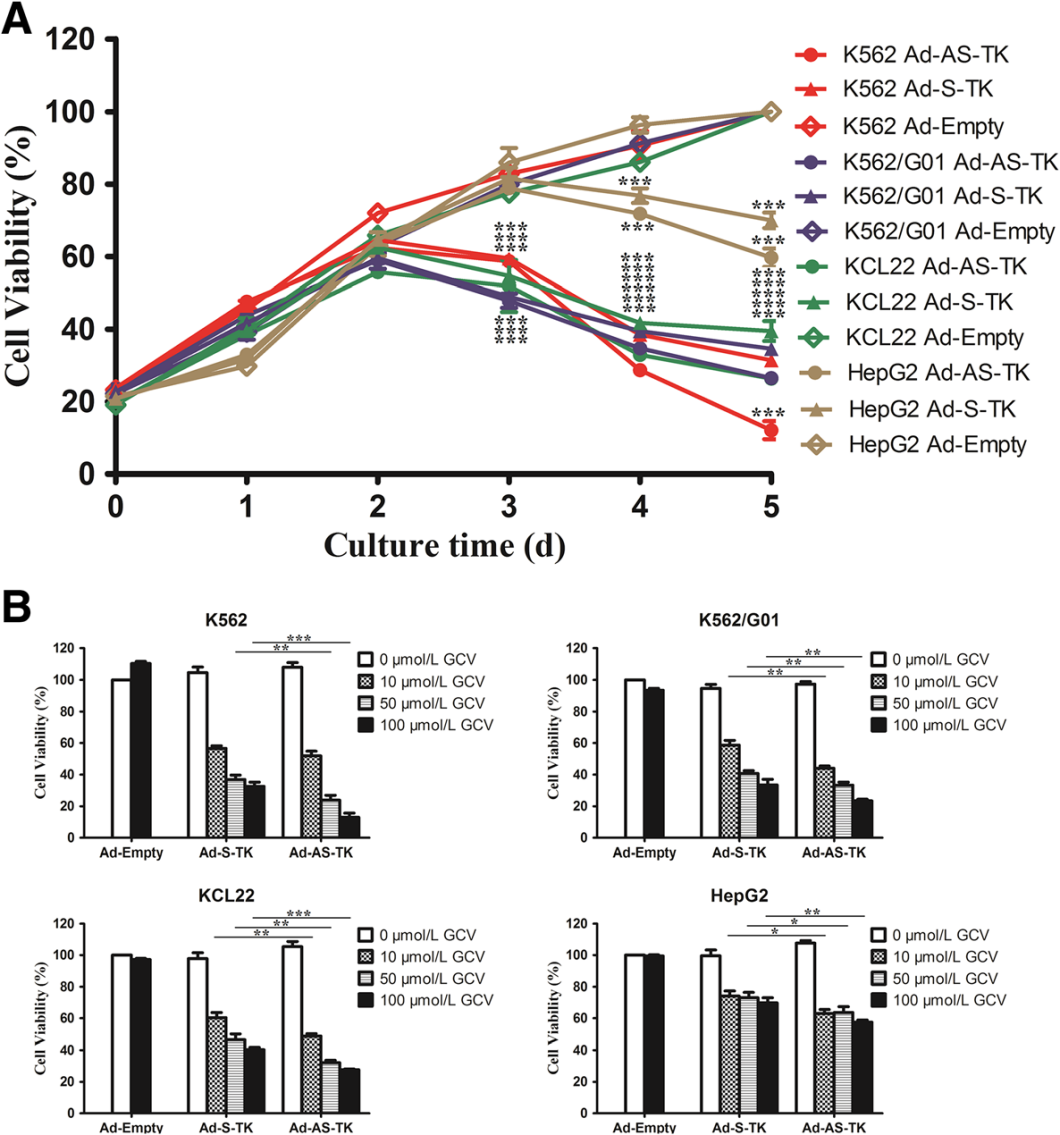
Cell survival assay of BP-CML cells with an ARE/SUZ12-regulated TK/GCV system. BP-CML cell lines and control cells were transduced with Ad-S-TK, Ad-AS-TK, or empty adenovirus, and cultured in different concentrations of GCV. Cell viability was assessed via CCK8 assay **(A)** with 100 μmol/l GCV treatment at d0, d1, d2, d3, d4, and d5 and **(B)** with 0–100 μmol/l GCV treatment at d5. *, P < 0.05; **, P < 0.01; ***, P < 0.001.

To measure viability of cells transduced with Ad-AS-TK/GCV, Ad-S-TK/GCV, or Ad-Empty/GCV, a WST assay was used. As shown in Figure 3, we observed that K562, KCL22, and K562/G01 cells treated with Ad-AS-TK/GCV were less viable compared to cells treated with Ad-S-TK/GCV (P <0.05). For example, absorbance of K562/G01 incubated with 10, 50, and 100 μmol/l of GCV was 0.44 (SD=0.02), 0.33 (SD=0.02) and 0.23 (SD=0.01) for Ad-AS-TK, and 0.59 (SD=0.03), 0.41 (SD=0.02) and 0.34 (SD=0.04) for Ad-S-TK, respectively, where reduction of cell viability by Ad-AS-TK/GCV were significantly more sharp (P <0.05). Loss of cell viability with Ad-AS-TK/GCV treatment suggested that Ad-AS-TK effectively killed BP-CML cells.

**Figure 4:**
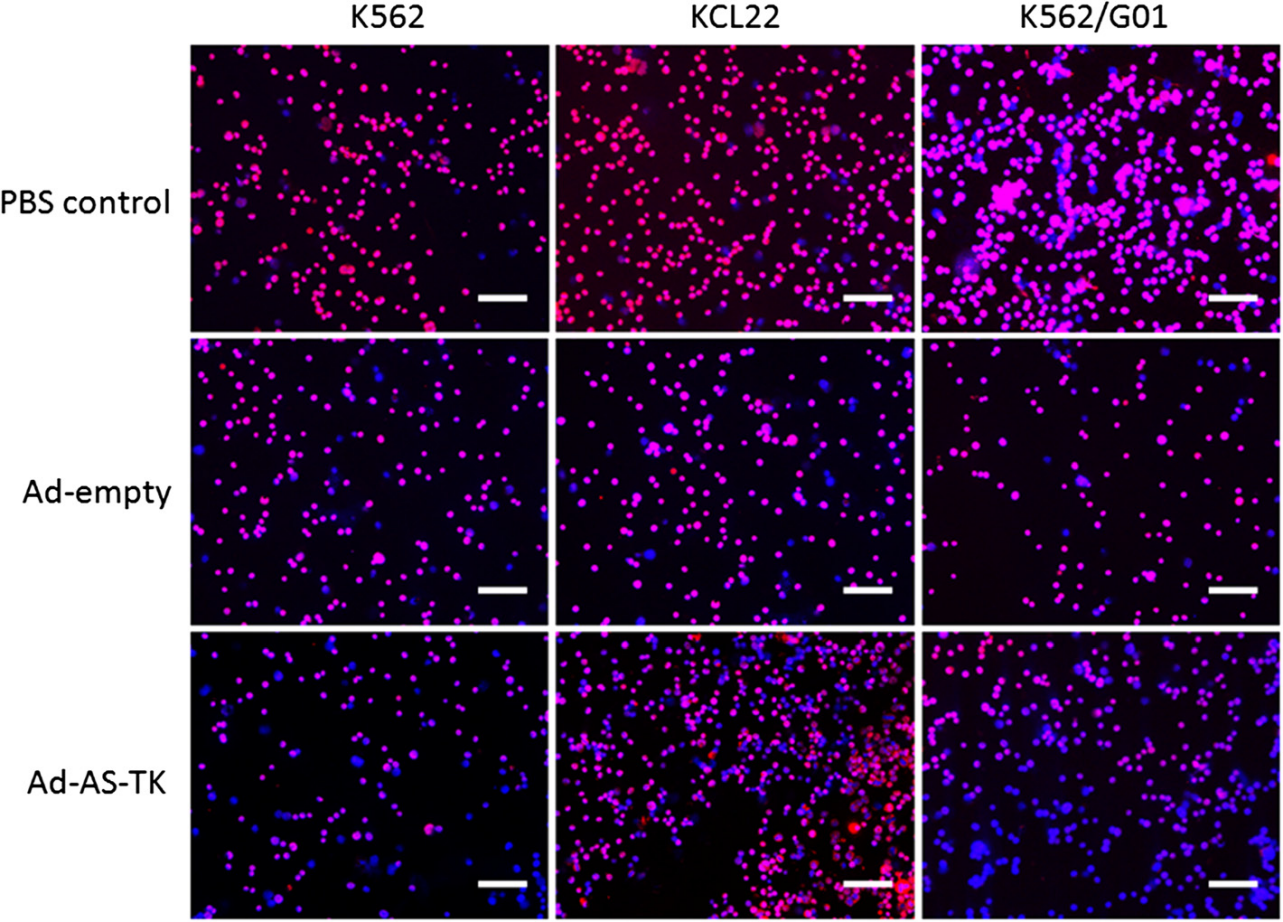
Immunofluorescent staining to measure SUZ12 in BP-CML cells with an ARE/SUZ12-regulated TK/GCV system. CML blast crisis cells were transduced with PBS, Ad-AS-TK, or empty adenovirus, and cultured in 100 μmol/l GCV for 48 h.

After incubating cells with Ad-AS-TK or Ad-S-TK and increasing concentrations of GCV (0–100 μmol/l), K562, KCL22, and K562/G01 cell viability progressively decreased. Both Ad-AS-TK/GCV and Ad-S-TK/GCV treatment showed significant GCV-dependent manner reduction in cell viability (Figure 3B). After cells were incubated in 100 μmol/l GCV and Ad-AS-TK for 5 days, K562, KCL22, and K562/G01 cells were almost eradicated. In contrast to BP-CML cells, HepG2 cell viability was only slightly affected after incubation with Ad-AS-TK or Ad-S-TK and 100 μmol/l GCV, and more than 60% of transduced HepG2 cells were viable (Figure 3A). An immunofluorescent assay with anti-SUZ12 antibody revealed that fewer than 50% Ad-AS-TK transduced BP-CML cells were SUZ12-positive, and more than 70% control cells were SUZ12-positive (Figure 4). The 2.3 kb exogenous SUZ12 fragment of Ad-AS-TK competes with the endogenous *SUZ12* gene for the same transcription factor binding, reducing endogenous SUZ12 promoter activity and SUZ12 expression.

### Apoptosis of BP-CML cells transduced with the ARE/SUZ12 dual-specific TK/GCV system

**Figure 5:**
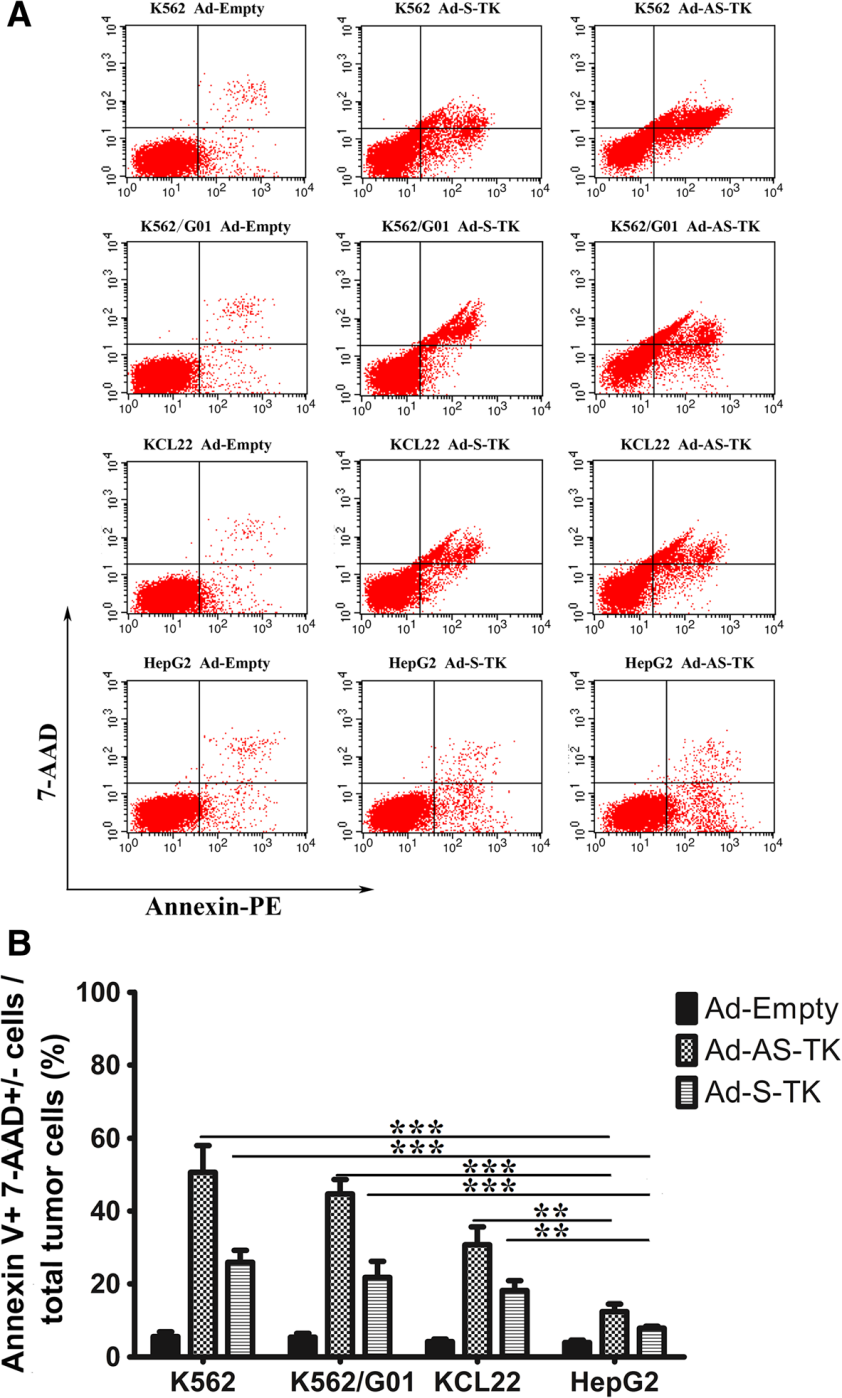
Apoptotic effects of BP-CML cells with an ARE/SUZ12-regulated TK/GCV system. BP-CML cell lines and control cells were transduced with Ad-S-TK, Ad-AS-TK, or empty adenovirus, and further cultured in 100 μmol/l GCV. The percentage of apoptotic cells was determined by annexin V–PE/7-AAD staining followed by 2-color flow cytometric analysis **(A)**. Data represent average percentage ± SEM, n=3. *, P < 0.05; **, P < 0.01; ***, P < 0.001 **(B)**.

To investigate whether Ad-AS-TK/GCV could exhibit enhanced apoptotic effects in BP-CML cells, the Ad transduced cells, treated with 100 μmol/l GCV for an additional 48 h, were assayed with Annexin V PE. The results showed that Ad-AS-TK was better at inducing cell apoptosis in ROS-signal activated BP-CML cells than Ad-S-TK (Figure 5A and B). After a 48 h incubation, apoptotic cells increased in Ad-AS-TK transfected cells compared to Ad-S-TK or empty adenovirus. k562 cells treated with Ad-AS-TK had the greatest degree of cell death (~51%), compared to k562 treated with Ad-S-TK (~28%). HepG2 cells treated with TK adenoviruses had the least amount of cell death (~12% for Ad-AS-TK, ~7% for Ad-S-TK). Thus, the Ad-AS-TK/GCV system can effectively and selectively induce apoptosis of BP-CML cells.

### Expression changes in cell cycle-related and apoptosis-related molecules

**Figure 6:**
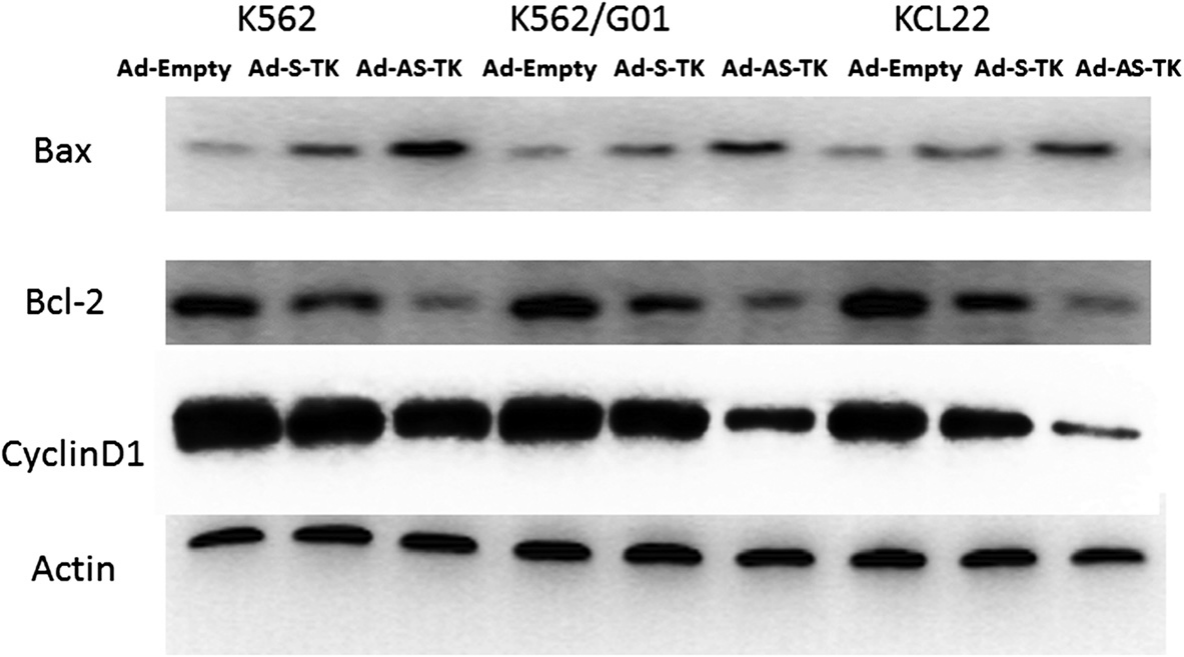
Expression of cycle-related and apoptotic molecules in BP-CML cells with an ARE/SUZ12-regulated TK/GCV system. Bcl-2, Cycline D1, and Bax. CML blast crisis cells were transduced with Ad-S-TK, Ad-AS-TK, or empty adenovirus, and cultured in 100 μmol/l GCV for 48 h. Expression of Bcl-2, Cyclin D1, and Bax analyzed with Western blot (actin internal control).

To test whether the Ad-AS-TK/GCV system altered expression of cycle-related and apoptosis-related proteins, we measured expression of Bcl-2, Cycline D1, and Bax. Figure 6 shows that BP-CML cells treated with the Ad-AS-TK/GCV system, compared with Ad-S-TK/GCV or empty adenovirus, had more Bax expression, and decreased Bcl-2 and Cycline D1 expression. Thus, the dually regulated Ad-AS-TK/GCV system could induce apoptosis or affect the cell cycle of BP-CML cells, compared with the Ad-S-TK/GCV system. These data agreed with Wright staining analysis (Additional file 1: Figure S1).

## Discussion

To date, gene therapy has shown promise for therapeutic applications but reported adverse events have caused concern in the scientific community about the safety of these approaches [20–22]. To improve safety, tightly regulated TK gene expression for blast-phase leukemia cells was conducted with a dual-specific composite promoter components in our study. Our data confirm the feasibility and efficacy of an ARE/SUZ12 dual-specific TK/GCV system for BP-CML gene therapy.

High constitutive expression of SUZ12 has been reported to drive proliferation, impair differentiation, and disrupt histone modification, which induces deregulation of transcriptional networks and aggressive leukemia. Pizzatti’s group reported that SUZ12 was overexpressed in BP-CML, and that this was due to SUZ12 promoter activation by constitutively activated WNT pathway molecules including catenin P120, WNT11, and WNT5A. Our data confirm a sharp increase in SUZ12 mRNA in BP-CML patient samples. Ridinge and colleagues reporeted that SUZ12 bound to the Bim promoter represses Bim pro-apoptotic factor transcription, thus inhibiting mitochondrial apoptosis of leukemia cells [23]. Meanwhile, Shi and coworkers reported that shRNA-mediated suppression of SUZ12 lead to proliferation arrest and initiation of differentiation of leukemia cells, and had minimal effects on the growth of several non-leukemia-transformed hematopoietic cell lines [24]. Similarly, Villa’ group revealed that knockdown of SUZ12 can revert not only histone modification but also DNA demethylation of PML-RARα target genes, contributing granulocytic differentiation in leukemic cells [25]. Therefore, the 2.3 kb upstream fragment harboring substantial promoter activity was measured with a luciferase assay and this fragment was cloned to regulate specific expression of the downstream *TK* gene in our study. To more tightly and strongly regulate TK expression, a combination of the ARE enhancer and the SUZ12 promoter was constructed.

Oxidative stress, an imbalance between the production and disposal of ROS, has been shown to be associated with CML progression and TKI resistance via oxidative DNA damage [26–30]. In response to oxidative stress, activated Nrf2 recognizes and binds to ARE of cytoprotective and detoxification genes, including NAD(P)H:quinone oxidoreductase (NQO1), and then Nrf2 stimulates transcriptional activation of genes in CML cells. We cloned the ARE of the *NQO1* gene and inserted this into upstream regulatory elements to regulate TK expression. Ad-AS-Luc had more luciferase activity in BP-CML cells, and Ad-AS-TK synergized ARE and the SUZ12 promoter for cell killing.

Due to basal promoter activity, apoptosis of BP-CML cells transduced with Ad-S-TK/GCV occurred, and the ARE/SUZ12 dual-specific Ad-AS-TK/GCV system conferred greater apoptosis of CML blast crisis cells. Likely tight and augmented TK regulation by ARE/SUZ12 composite components may help eradicate leukemia cells. ARE performed a specific enhancer role that ARE enhanced the translation of the downstream *TK* gene in CML cell. Also, the 2.3 kb exogenous SUZ12 fragment of Ad competes with the endogenous *SUZ12* gene for the same transcription factor binding, causing SUZ12 expression loss (Figure 4) and cell proliferation arrest [24]. Third, Nrf2 functions to activate translation of cytoprotective genes and protect cells from electrophilic and oxidative damage in normal cells. However, in cancerous cells, Nrf2 is pro-tumor as the same cytoprotective genes protect human leukemia cells from apoptotic signals and enhance cancer cell chemo-resistance [31]. The exogenous ARE of the Ad competes with the endogenous ARE of cytoprotective genes for Nrf2, thus at least partly blocking endogenous Nrf2 signaling pathways of leukemia cells. However, our immunofluorescent assay with anti-NQO1 antibody did not confirm significant differences in NQO1 expression in cells incubated with Ad-AS-TK or Ad-Empty (data not shown). Therefore, therapeutic efficacy was enhanced via synergism of ARE/SUZ12 specific regulation. This suggests that Ad-AS-TK may be beneficial, safe, and efficacious for blast crisis CML.

Blast crisis as first presentation of CML accounts for 10% of all CML cases [32], with a median survival of less than 6 months, while the rest 90% cases diagnosed with CP-CML have been potentially in process to blast crisis. Median survival of CP-CML is estimated at a median of 25 to 30 years, and the blastic transformation rate has estimated at 1% to 1.5% per year in the imatinib era, which means 25% to 45% CP-CML would be into blast phase [33,34]. Nowadays, CML patients have multiple treatment options, including five TKIs (imatinib, nilotinib, dasatinib, bosutinib, and ponatinib), omace-taxine (protein synthesis inhibition), and several older agents (hydroxurea, interferon alpha, busulfan, 6-mercaptopurine, cytarabine, decitabine, etc.). TKIs monotherapy or in combination with chemotherapy may serve as a preferred option for BP-CML, and provide a median survival of 9–12 months in BP CML. As a novel way of treatment, gene therapy bring a new hope for BP CML patients. As a potential treatment strategy of BP-CML, there is still a question whether Ad-AS-TK/GCV system is efficacious for primary leukemic cells of all the BP-CML patients. In our study, an adenoviral type 5 (Ad5) vector was introduced into the systemic delivery of modified AS-TK construct, and the effect of Ad-AS-TK/GCV system is directly associated with adenoviral gene transfer efficiency into hematopoietic cells. Efficient gene transfer with Ad5 vectors generally depends on the initial attachment of their fiber, which binds the coxsackie–adenovirus receptor (CAR) of cells, and their subsequent internalization. Primary leukemic cells of some CML patients lack CAR and are therefore resistant to Ad5 transduction, gene transfer efficiency of Ad5 can differ greatly among individual CML patient cells [35] (gene transfer rates of RGD-Ad5 can be 30 ~ 80%), which means different apoptosis effect of Ad-AS-TK/GCV system for different CML patient cells. Currently, integrating vectors derived from retroviruses and lentiviruses remain a preferred choice for their preferable efficiency in haematological clinical trials, however, integration of exogenous gene quasi-randomly throughout the human genome has been reported to cause an alarming incidence of vector-induced malignancy [36]. The stable, safe and effective gene transfer strategies should be further exploited to deliver exogenous gene into hematopoietic cells to overcome technology bottleneck of clinical leukemia gene therapy.

## Conclusions

In the study, we constructed an ARE/SUZ12 dual-specific TK/GCV system for blast phase leukemia gene therapy. The system was composed of two regulatory elements, an ARE for oxide stress regulation and a SUZ12 promoter for specific promoter activity in BP-CML cells. Our work suggests the feasibility of an ARE/SUZ12-regulated TK/GCV system for selectively eliminating BP-CML cells, suggesting that suicide gene engineering may be an effective strategy for treating BP-CML.

## Additional file

Additional file 1: Figure S1. Apoptosis analysis with Wright’s staining in BP-CML cells with an ARE/SUZ12-regulated TK/GCV system. CML blast crisis cells were transduced with Ad-S-TK, Ad-AS-TK, or empty adenovirus, and cultured in 100 μmol/l GCV for 48 h. Wright staining analysis of K562 (A), K562/G01 (B), KCL22 (C) for different groups.

## Abbreviations

Ad: adenoviral
alloSCT: Allogeneic stem cell transplantation
ARE: Antioxidant response element
BP: Blast Crisis phase
BP-CML: Blast phase chronic myeloid leukemia
CAR: Coxsackie–adenovirus receptor
CML: Chronic myeloid leukemia
CP: Chronic phase
GCV: Ganciclovir
GVHD: Graft-versus-host disease
HSV-Tk: Herpes simplex virus thymidine kinase gene
Nrf2: Nuclear factor E2-related factor 2
OS: Overall survival
PPC2: Polycomb repressive complex 2
TKIs: Tyrosine kinase inhibitors
