# Correction to: ARE/SUZ12 dual specifically-regulated adenoviral TK/GCV system for CML blast crisis cells

**DOI:** 10.1186/s13046-022-02279-4

**Published:** 2022-02-09

**Authors:** Bailing Zu, Yi Shi, Min Xu, Guoling You, Zhenglan Huang, Miao Gao, Wenli Feng

**Affiliations:** 1 Department of Clinical Hematology, Key Laboratory of Laboratory Medical Diagnostics of Ministry of Education, Chongqing Medical University, No.1, Yixueyuan Road, Chongqing, 400016 People’s Republic of China; 2 Department of Clinical Laboratory, Shanghai Pudong Hospital, Fudan University Pudong Medical Center, Shanghai, China; 3 Department of Clinical Laboratory, Shanghai Children’s Medical Center, Shanghai Jiaotong University School of Medicine, Shanghai, China

**Correction to: J Exp Clin Cancer Res 34, 56 (2015)**

**https://doi.org/10.1186/s13046-015-0139-4**

Following publication of the original article [1], the authors identified a minor error in Figure 4, specifically:

- Fig. 4: incorrect immunofluorescent staining image used to represent KCL22 Ad-empty control (2^nd^ row, 2^nd^ column); the image has been replaced with the correct image

The corrected figure is given here. The correction does not have any effect on the final conclusions of the paper. 

**Fig. 4:**
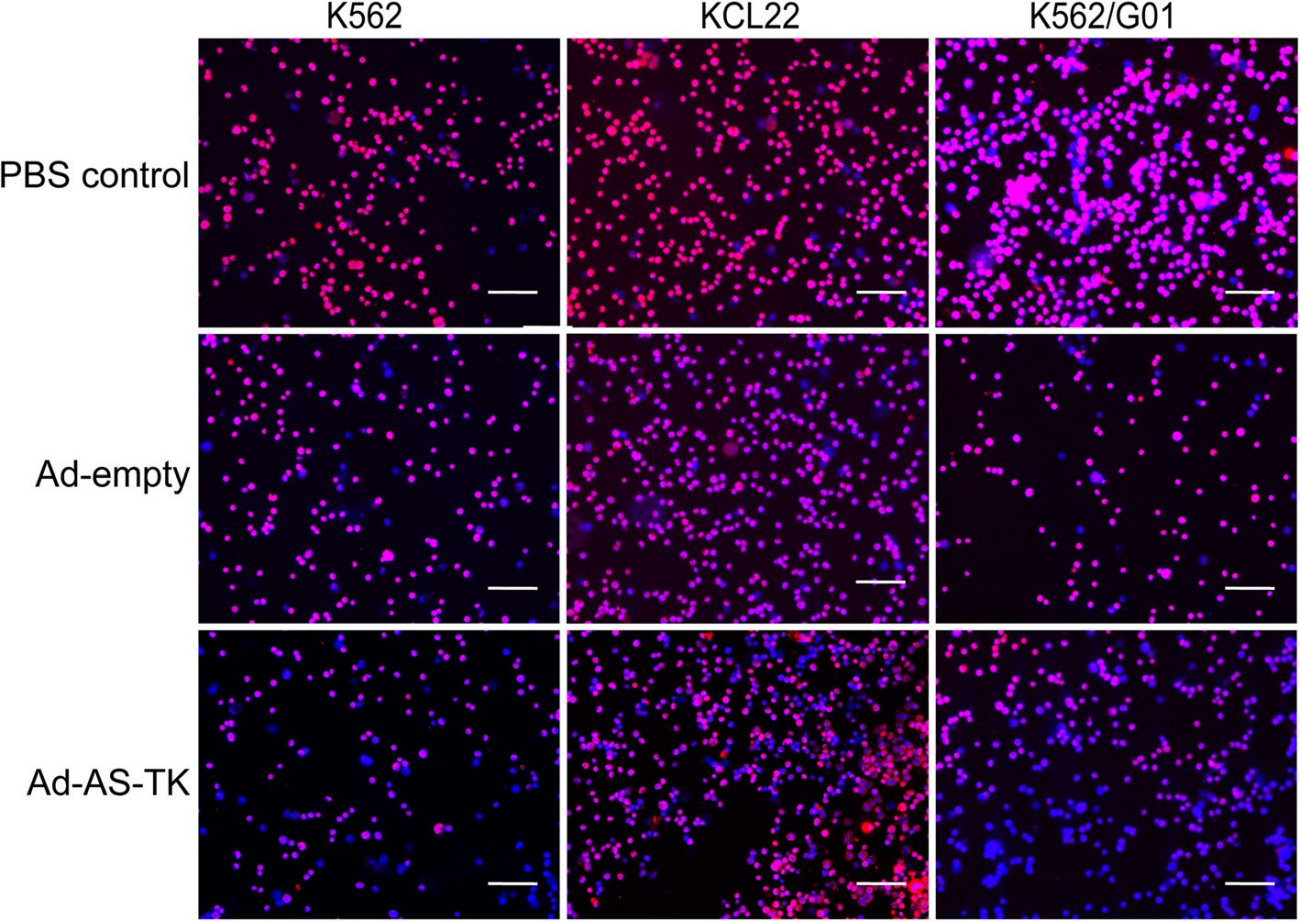
Immunofluorescent staining to measure SUZ12 in BP-CML cells with an ARE/SUZ12-regulated TK/GCV system. CML blast crisis cells were transduced with PBS, Ad-AS-TK, or empty adenovirus, and cultured in 100 μmol/l GCV for 48 h
