# Supplementary figures and images for: ARE/SUZ12 dual specifically-regulated adenoviral TK/GCV system for CML blast crisis cells
Source: J Exp Clin Cancer Res. 2015 May 28;34(1):56. doi: 10.1186/s13046-015-0139-4 (PMC4456766; doi:10.1186/s13046-015-0139-4)

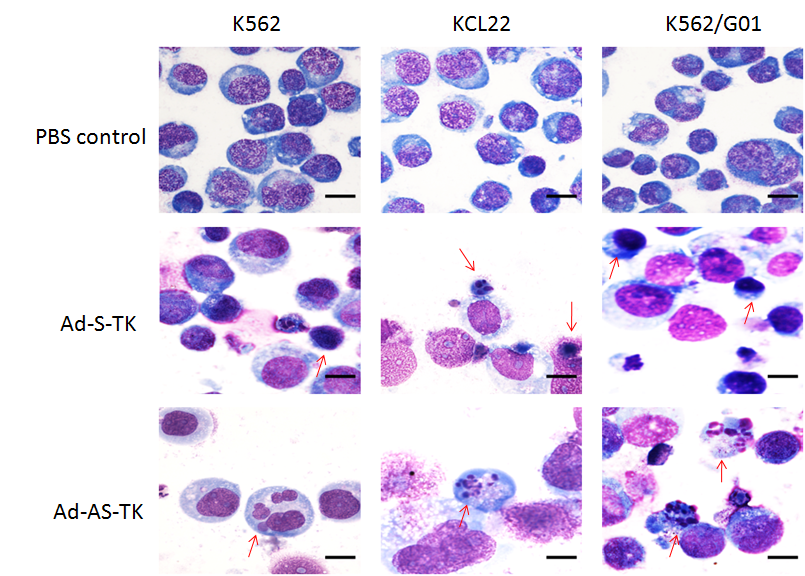

Supplement: Additional file 1: Figure S1. — Apoptosis analysis with Wright’s staining in BP-CML cells with an ARE/SUZ12-regulated TK/GCV system. CML blast crisis cells were transduced with Ad-S-TK, Ad-AS-TK, or empty adenovirus, and cultured in 100 μmol/l GCV for 48 h. Wright staining analysis of K562 (A), K562/G01 (B), KCL22 (C) for different groups. [file 13046_2015_139_MOESM1_ESM.tiff]
